# MBOAT7 rs641738 (C>T) is associated with NAFLD progression in men and decreased ASCVD risk in elder Chinese population

**DOI:** 10.3389/fendo.2023.1199429

**Published:** 2023-06-22

**Authors:** Xiaoyi Xu, Hangfei Xu, Xiaohui Liu, Shuang Zhang, Zhenhuan Cao, Lixia Qiu, Xiaofei Du, Yali Liu, Gang Wang, Li Zhang, Yang Zhang, Jing Zhang

**Affiliations:** ^1^ The Third Unit, The Department of Hepatology, Beijing Youan Hospital, Capital Medical University, Beijing, China; ^2^ Beijing Institute of Hepatology, Beijing Youan Hospital, Capital Medical University, Beijing, China; ^3^ Menkuang Hospital, Beijing Jingmei Group General Hospital, Beijing, China

**Keywords:** MBOAT7, non-alcoholic fatty liver disease, fibrosis, atherosclerotic cardiovascular disease, single-nucleotide polymorphism

## Abstract

**Background and aim:**

The MBOAT7 rs641738 (C>T) variant has demonstrated an association with non-alcoholic fatty liver disease (NAFLD) in both adult and pediatric patients, while few studies have been conducted in elderly populations. Hence, a case–control study was undertaken to assess their correlation in elderly residents in a Beijing community.

**Materials and methods:**

A total of 1,287 participants were included. Medical history, abdominal ultrasound, and laboratory tests were recorded. Liver fat content and fibrosis stage were detected by Fibroscan. Genotyping of genomic DNA was performed using the 96.96 genotyping integrated fluidics circuit.

**Results:**

Of the recruited subjects, 638 subjects (56.60%) had NAFLD, and 398 subjects (35.28%) had atherosclerotic cardiovascular disease (ASCVD). T allele carriage was associated with higher ALT (p=0.005) and significant fibrosis in male NAFLD patients (p=0.005) compared to CC genotype. TT genotype was associated with reduced risk of metabolic syndrome (OR=0.589, 95%CI: 0.114–0.683, p=0.005) and type 2 diabetes (OR=0.804, 95%CI: 0.277–0.296, p=0.048) in NAFLD population when compared to the CC genotype. In addition, TT genotype was also associated with reduced risk of ASCVD (OR=0.570, 95%CI:0.340–0.953, p=0.032) and less obesity (OR=0.545, 95%CI: 0.346–0.856, p=0.008) in the whole population.

**Conclusion:**

MBOAT7 rs641738 (C>T) variant was associated with fibrosis in male NAFLD patients. The variant also reduced risk of metabolic traits and type 2 diabetes in NAFLD and ASCVD risk in Chinese elders.

## Introduction

Given that the prevalence of non-alcoholic fatty liver disease (NAFLD) covers higher than 25% of the population, it has become the most common chronic liver disease worldwide (1, 2). The onset of NAFLD is characterized by an imbalance in lipid metabolism, leading to the accumulation of lipid droplets in hepatocytes, a condition that progresses to non-alcoholic steatohepatitis, fibrosis, cirrhosis, and ultimately hepatocellular carcinoma (HCC) (3–5). Obesity, type 2 diabetes (T2DM), dyslipidemia, hypertension, and cardiovascular disorders, etc. were linked strongly to NAFLD (6, 7). Over the last decade, there has been growing interest on the role of single nucleotide polymorphisms (SNPs) in the progression of NAFLD. Multiple SNPs, including patatin-like phospholipase domain-containing protein 3 (PNPLA3, rs738409), transmembrane 6 superfamily member 2 (TM6SF2, rs58542926), etc., were found to be closely related to the incidence and progression of NAFLD (8, 9). Furthermore, they were also associated with reduced risk of atherosclerotic cardiovascular disease (ASCVD), which is the major cause of death among NAFLD patients (10–13). The effect of SNPs deepened our understanding of NAFLD and the relationship between NAFLD and cardio-metabolic disease.

Membrane bound O-acyltransferase domain containing 7 (MBOAT7) is a multi-transmembrane protein and an important member of the “Lands’ Cycle” of membrane phosphatidyl chain remodeling (14). In 2015, a genome-wide association study identified that the genetic variant near MBOAT7 (rs641738 C>T) was a risk factor for alcohol-related cirrhosis (15). Subsequently, studies conducted on NAFLD/NASH revealed that MBOAT7 rs641738 C> T was positively related to hepatic fat content and histological severity (16) only in European descent (17). Loss or variant of MBOAT7 reduced liver mRNA and protein expression and consequently led to changes in phosphorylated inositol species, which potentially contributed to liver injury (16, 18).

The clinical significance of MBOAT7 rs641738 C>T variant on NAFLD has been evaluated in various races and age groups; however, its impact on the elderly population remained unclear. In the current study, the relationship between MBOAT7 rs641738 variant with the risk of NAFLD and hepatic fibrosis in an elderly population. Meanwhile, the correlation of the rs641738 T allele with metabolic traits and ASCVD was also analyzed. Furthermore, we explored the effect of rs641738 variant on serum ANGPTL3 level in order to explain the link between MBOAT7 with ASCVD.

## Materials and methods

### Patient cohort

Elder citizens in Beijing Mentougou community who participated in annual free physical examination was recruited from 1/11/2020 to 30/9/2021. The study protocol was approved by the individual Ethics Review Committee of Beijing Youan Hospital (IRB approval number [2020]-233). All study subjects provided written informed consent.

The study was carried out in Menkuang Hospital, Beijing Jingmei Group General Hospital. The inclusion criteria were (1) patients with fatty liver diagnosed by ultrasound and (2) who signed informed consent. The exclusion criteria were (1) excessive alcohol consumption (≥30g/day in men or 20 g/day in women); (2) lack of medical history or lab results; (3) inability to obtain reliable abdominal ultrasound results due to specific reasons, such as intestinal gas interference; (4) malignant tumors, HIV, and other serious diseases that may affect the nutritional status or organ function; and (5) comorbidity with other liver diseases, such as viral hepatitis and autoimmune hepatitis. The controlled groups were residents without fatty liver examined by ultrasound.

**Figure 1:**
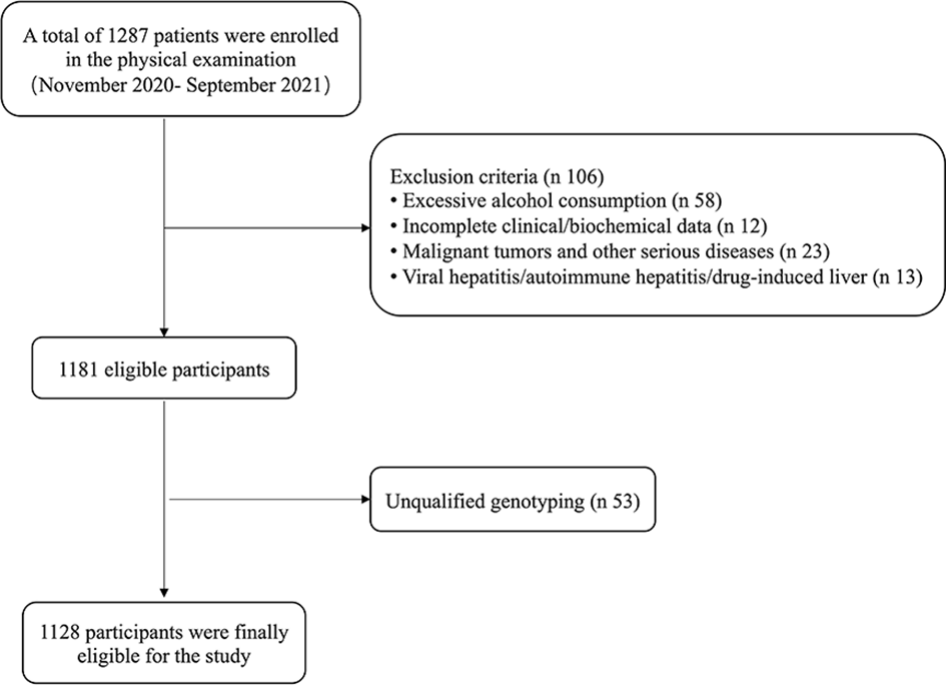
Flow chart of patients’ selection.

Usually, 10–20 residents took part in the routine examination every morning. If a patient is diagnosed with fatty liver by ultrasound, he/she is transferred to an isolated room to be recruited and further examined. The next resident without fatty liver following the previous NAFLD patients was recruited as control. Eventually, 1,287 residents were examined, and 1,128 residents were recruited in the study (**Figure 1**).

### Data collection

The routine free examination included abdomen ultrasound examination, routine blood test, routine urine test, liver function, renal function, fasting blood sugar (FBS), glycosylated hemoglobin (HbA1c), and lipid profile. Extra blood sample was obtained and tested for hypersensitive C-reactive protein (hs-CRP) and fasting insulin (FINS) in addition to the routine examination. All laboratory tests were carried out in the central lab of Menkuang Hospital or Beijing Jingmei Group General Hospital.

Two trained investigators carried out the whole study. They were responsible for recruiting patients, performing anthropometric examinations, and determining liver fat content and liver stiffness (LSM) by FibroScan 502 touch device (Echosens, Paris, France). The demographic indicators and medical histories were extracted from the community health records and checked again on site.

### Definitions

Fatty liver was diagnosed by ultrasound, and participants were divided into two groups, NAFLD and non NAFLD. Body mass index (BMI) was calculated as weight (kg) divided by the square of the height (m). Waist-to-hip ratio (WHR) was calculated as waist circumference divided by hip circumference. Homeostasis model assessment insulin resistant (HOMA-IR) was calculated as (FINS (pmol/L) × FBS (mmol/L))/22.5. Additionally, non-invasive liver fibrosis score formulas fibrosis-4 index (FIB-4) (19) and AST-to-platelet ratio index (APRI) (20) were calculated according to the following formulas:


$$\text{FIB}-4=\text{age}\left[\text{years}\right]\times \text{AST}\left[\text{IU}/\text{L}\right]/\text{platelet count}\left[\times \;10^{9}/\text{L}\right]\times \left(\text{ALT}1/2\left[\text{IU}/\text{L}\right]\right)$$

$$\text{APRI}=\text{AST}\left[\text{IU}/L\right]\times 100/\text{platelet count}\left[\times \;10^{9}/L\right]$$

The comorbid diseases were diagnosed according to international guidelines, including hypertension, ASCVD, T2DM, and metabolic syndrome (MetS). In brief, hypertension is diagnosed when systolic blood pressure ≥130mmHg or diastolic blood pressure ≥85mmHg or when taking antihypertensive drugs. T2DM is diagnosed when FBS ≥7.0 mmol/L, HbA1c ≥ 6.5%, OGTT 2 h blood sugar ≥ 11.1mmo/L, or taking hypoglycemic drugs. MetS was defined in accordance with the criteria, which was based on the presence of at least three of the following components: (1) elevated waist circumference (≥90cm for men or ≥80 cm for women); (2) elevated triglycerides (≥1.70 mmol/L) or drug treatment for elevated triglycerides; (3) reduced HDL-C (<1.0 mmol/L for men and<1.3 mmol/L for women) or drug treatment to reduce HDL-C; (4) elevated blood pressure (≥ 130/85 mmHg) or drug treatment for hypertension; and (5) elevated FBS (≥5.6 mmol/L) or drug treatment for elevated glucose(21). ASCVD was diagnosed when coronary angiography (CAG) showed that the left main artery, left anterior descending artery, left circumflex artery, right coronary artery, or any one or more of its main branches had stenosis of ≥50% (22) or history of myocardial infarction. In this study, LSM ≥8.2 kPa and APRI ≥0.5 were used to predict significant hepatic fibrosis (23, 20). Obesity was defined as BMI≥25 kg/m^2^ (24).

### Genomic DNA extracting and genotyping

Genomic DNA was extracted from the patient’s blood specimens (BGI-Shenzhen, China). DNA concentration and quality were determined by spectrophotometry (Nanodrop 2000, Thermo Scientific, Wilmington, DE) and standardized to approximately 50 ng/ml before genotyping. Next, genomic DNA has genotyped with the usage of a 96.96 genotyping integrated fluidics circuit (IFC) with customized SNP-type assays on the Juno™ system (Fluidigm, South San Francisco, CA, USA) and quantification on the Biomark™ (Fluidigm, South San Francisco, USA) in accordance with the manufacturers’ instructions. All genotyping was blinded to clinical variables. The Fluidigm SNP Genotyping Analysis program, version 4.5.1, was used to analyze the data (South San Francisco, CA, USA).

### Serum ANGPTL3 determination by ELISA

Serum ANGPTL3 levels were determined in 120 patients who were randomly selected from subjects with the three genotypes, each group containing 40 patients. ANGPTL3 was quantified by ELISA according to the instructions (Cusabio, Wuhan, China). The intra- and inter-assay coefficients of variation of ANPTL3 were<8% and 10%, respectively.

### Statistical analysis

Clinical characteristics were compared between the rs641738 T allele carriage and non-T carriage or among the three genotypes. Descriptive values were expressed as mean ± standard deviation (SD) or medians and interquartile ranges (IQRs) depending on the distribution of the data. Student’s t-test or Mann–Whitney–Wilcoxon test was used to assess continuous variables according to value distributions. Frequencies and percentages were used to summarize categorical variables, and data were compared by using Pearson’s chi-square when appropriate. The χ^2^ test was also used if indicated. All comparison tests between the two groups were two-tailed with a 95% confidence interval. Univariate and multivariate logistic regression analyses were performed to assess the independent predictors for NAFLD, ASCVD, hypertension, and MetS. The statistical significance was set at p-value<0.05. The Statistical Package for Social Science for Windows, version 19.0 (SPSS Inc., IBM, New York), GraphPad Prism (version 2.0), and R language (version 3.4.3) were used for data analyses.

## Results

### Clinical characteristics in NAFLD and non-NAFLD group

A total of 1,128 subjects were enrolled in the study. Characteristics of the NAFLD and non-NAFLD populations are provided in **Table 1**. Among them, 638 subjects (56.60%) had NAFLD, 469 subjects (41.58%) had T2DM, 869 subjects (77.04%) had hypertension, 948 subjects (84.93%) had MetS, and 398 subjects (35.28%) had ASCVD. When compared to the non-NAFLD subjects, NAFLD patients were likely to be elderly, female, with higher weight, BMI, waist circumference, and WHR. In addition, these patients exhibited significantly higher CAP, LSM, ALT, AST, TG, FINS, FBS, HOMA-IR, FIB-4, and APRI, and lower HDL (**Table 1**). However, the serum levels of TC and LDL and the prevalence of ASCVD were comparable between the NAFLD and non-NAFLD groups. The distribution of MBOAT7 rs641738 polymorphism in NAFLD and non-NAFLD groups was consistent with Hardy–Weinberg balance and was representative of the population (p>0.05).

**Table 1:** Clinical characteristics of NAFLD and non-NAFLD patients.

	Total (N=1128)	Non-NAFLD (N=490)	NAFLD (N=638)	p-value
Age (year)	69.00 (67.00–74.00)	70.00 (67.00–75.00)	69.00 (67.00–73.00)	**<0.001**
Male, n (%)	316 (28.01)	166 (33.88)	150 (23.51)	**<0.001**
Weight (kg)	64.65 (58.50–71.93)	61.70 (55.90–67.95)	67.30 (60.95–74.45)	**<0.001**
BMI (kg/m^2^)	25.60 (23.70–27.83)	24.40 (22.50–26.00)	26.80 (24.70–28.80)	**<0.001**
Waist circumference (cm)	88.00 (83.00–94.00)	85.00 (80.00–90.00)	90.00 (85.00–96.00)	**<0.001**
Hip circumference (cm)	98.00 (94.00–103.00)	95.00 (90.00–100.00)	100.00 (95.00–105.00)	**<0.001**
WHR	0.90 (0.88–0.93)	0.89 (0.87–0.92)	0.90 (0.88–0.93)	**<0.001**
ASCVD, n (%)	398 (35.28)	177 (36.10)	221 (34.60)	0.605
Hypertension, n (%)	869 (77.04)	357 (72.90)	512 (80.30)	**0.003**
MetS, n (%)	958 (84.93)	371 (75.70)	587 (92.00)	**<0.001**
T2DM, n (%)	469 (41.58)	176 (35.90)	293 (45.90)	**0.001**
Obesity, n (%)	660 (58.51)	196 (39.00)	464 (72.70)	**<0.001**
Lipid lowering agent, n (%)	388 (34.40)	172 (35.10)	216 (33.86)	0.662
ALT (U/L)	18.00 (14.00–23.00)	16.00 (13.00–21.00)	19.00 (15.00–25.00)	**<0.001**
AST (U/L)	17.00 (14.00–21.00)	17.00 (14.00–20.00)	18.00 (15.00–22.00)	**0.001**
TG (mmol/L)	0.97 (1.36–1.90)	1.16 (0.84–1.62)	1.56 (1.12–2.06)	**<0.001**
TC (mmol/L)	4.70 (4.01–5.52)	4.69 (4.01–5.56)	4.75 (4.00–5.50)	0.985
HDL (mmol/L)	1.13 (0.98–1.30)	1.19 (1.00–1.37)	1.11 (0.96–1.24)	**<0.001**
LDL (mmol/L)	3.23 (2.58–3.99)	3.21 (2.59–3.97)	3.24 (2.58–4.00)	0.821
FBS (mmol/L)	6.26 (5.69–7.42)	6.03 (5.60–7.08)	6.49 (5.86–7.78)	**<0.001**
FINS (mIU/L)	9.34 (6.30–13.61)	7.65 (5.39–11.05)	11.19 (7.89–15.80)	**<0.001**
HOMA-IR	2.76 (1.76–4.24)	2.16 (1.45–3.41)	3.39 (2.33–5.02)	**<0.001**
HbA1c (%)	6.10 (5.80–6.90)	6.00 (5.70–6.53)	6.20 (5.90–7.10)	**<0.001**
CAP (dB/m)	275.00 (239.00–311.00)	241.0 (215.00–266.50)	298.00 (270.00–324.25)	**<0.001**
LSM (kPa)	4.60 (3.80–5.80)	4.10 (3.50–5.10)	5.00 (4.10–6.30)	**<0.001**
FIB-4	1.33 (1.04–1.69)	1.39 (1.12–1.77)	1.28 (0.98–1.62)	**<0.001**
APRI score	0.20 (0.15–0.26)	0.18 (0.15–0.24)	0.21 (0.15–0.28)	**0.001**
MBOAT7 rs641738				0.467
CC	605 (53.63)	272 (55.51)	333 (52.19)	
CT	435 (38.56)	179 (36.53)	256 (40.13)	
TT	88 (7.80)	39 (7.96)	49 (7.68)	

BMI, body mass index; WHR, Waist-to-hip ratio; ASCVD, atherosclerotic cardiovascular disease; MetS, metabolic syndrome; T2DM, type 2 diabetes; ALT, alanine aminotransferase; AST, aspartate aminotransferase; TG, total triglyceride; TC, total cholesterol; HDL, high‐density lipoprotein; LDL, low‐density lipoprotein; FBS, fast blood sugar; FINS, fast insulin; HOMA‐IR, homoeostatic model assessment of insulin resistance; CAP, controlled attenuated parameter controlled; LSM, liver stiffness measurement; FIB‐4, fibrosis‐4 index; APRI, AST platelet ratio index.

Bold: p< 0.05.

### Comparison of characteristics according to MBOAT7 rs641738 genotype within NAFLD cohorts

**Figure 2:**
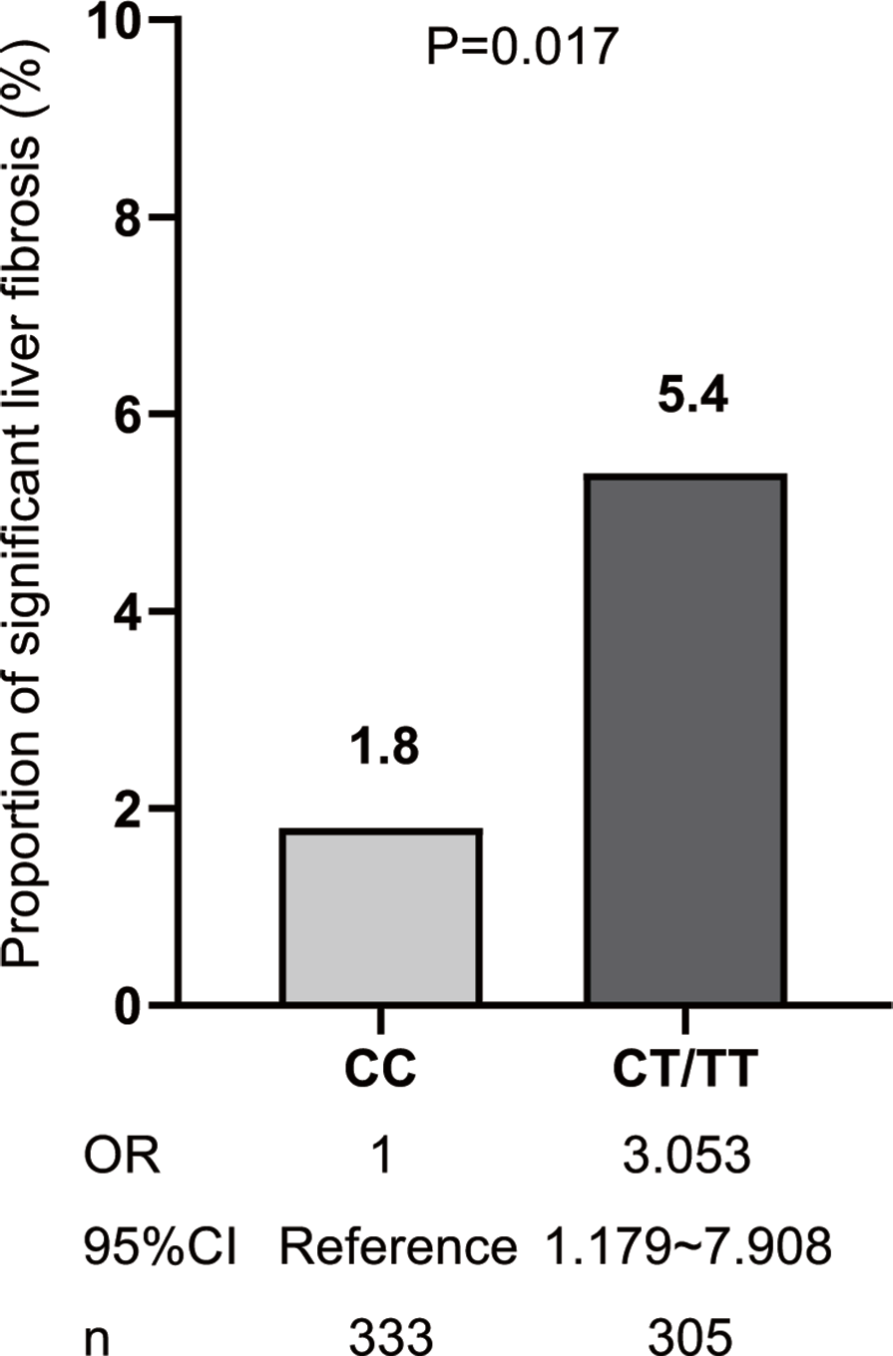
The risk of significant fibrosis for MBOAT7 rs641738 T allele carriage. OR, CT/TT versus CC genotype.

Among the NAFLD patients, carriage of the rs641738 C >T polymorphism accounted for 47.81% (305/638). These patients presented significantly higher TC, HDL, LDL, and FINS than non-T carriage (p<0.05, **Table 2**). After adjusting usage of lipid-lowering agents, TC and LDL levels were comparable between T carriage and non-T carriage groups, while HDL is still statistically significant (OR: 2.476, 95% CI:1.218–4.998, p=0.012). T allele carriage was also significantly associated with advanced fibrosis (5.4% versus 1.8%, OR: 3.053, 95% CI: 1.179–7.908, p=0.022, **Figure 2**). Even after adjusting for confounders, such as age, sex, and BMI, the risk of significant liver fibrosis remained statistically significant (OR: 3.024, 95% CI: 1.165–7.848, p=0.023).

**Table 2:** Comparison of clinical characteristics according to MBOAT7 rs641738 genotypes within NAFLD cohorts.

NAFLD	CC (N=333)	CT+TT (N=305)	p-value
Age (year)	69.00 (66.00–72.00)	69.00 (67.00–73.00)	0.279
Male, n (%)	79 (23.72)	71 (23.28)	0.895
Weight (Kg)	67.30 (60.80–74.50)	67.05 (61.10–74.40)	0.927
BMI (Kg/m2)	26.50 (24.50–28.80)	27.00 (25.03–28.80)	0.237
Waist circumference (cm)	90.00 (85.00–97.00)	90.00 (85.00–95.00)	0.540
Hip circumference (cm)	100.00 (95.00–105.50)	100.00 (95.00–105.00)	0.104
Waist-to-hip ratio	0.90 (0.88–0.93)	0.90 (0.89–0.94)	0.140
ASCVD, n (%)	116 (34.90)	106 (34.60)	0.983
Hypertension, n (%)	273 (82.00)	239 (78.40)	0.251
MetS, n (%)	313 (94.00)	274 (89.80)	0.053
T2DM, n (%)	159 (47.70)	134 (43.90)	0.334
Obesity, n (%)	233 (70.0)	231 (75.70)	0.102
Lipid lowering agent, n (%)	124 (37.23)	92 (30.16)	0.059
ALT (U/L)	19.00 (15.00–25.00)	20.00 (16.00–27.00)	0.091
AST (U/L)	18.00 (14.00–22.00)	18.00 (15.00–22.00)	0.869
TG (mmol/L)	1.56 (1.12–2.05)	1.54 (1.14–2.06)	0.876
TC (mmol/L)	4.59 (3.87–5.38)	4.86 (4.14–5.63)	**0.025**
HDL (mmol/L)	1.09 (0.95–1.23)	1.12 (0.98–1.26)	**0.031**
LDL (mmol/L)	3.15 (2.50–3.90)	3.67 (2.70–4.09)	**0.034**
hs-CRP (mg/L)	1.50 (0.80–2.60)	1.60 (0.91–2.93)	0.198
FBS (mmol/L)	6.50 (5.90–7.85)	6.49 (5.84–7.74)	0.465
FINS (mIU/L)	10.67 (7.51–14.04)	11.62 (8.43–18.11)	**0.016**
HOMA-IR	3.25 (2.27–4.51)	3.54 (2.47–5.93)	0.069
HbA1c (%)	6.30 (5.90–7.30)	6.20 (5.90–7.00)	0.165
CAP (dB/m)	295.43 ± 40.97	297.54 ± 40.85	0.542
LSM (kPa)	5.00 (4.10–6.10)	5.00 (4.10–6.40)	0.826
≥8.2 Kpa, n (%)	30 (9.00)	31 (10.30)	0.620
FIB-4	1.29 (1.01–1.67)	1.24 (0.93–1.60)	0.475
APRI score	0.20 (0.15–0.27)	0.19 (0.15–0.27)	0.686
>0.5, n (%)	6 (1.80)	16 (5.40)	**0.017**

BMI, body mass index; WHR, Waist-to-hip ratio; ASCVD, atherosclerotic cardiovascular disease; MetS, metabolic syndrome; T2DM, type 2 diabetes; ALT, alanine aminotransferase; AST, aspartate aminotransferase; TG, total triglyceride; TC, total cholesterol; HDL, high‐density lipoprotein; LDL, low‐density lipoprotein; FBS, fast blood sugar; FINS, fast insulin; HOMA‐IR, homoeostatic model assessment of insulin resistance; CAP, controlled attenuated parameter controlled; LSM, liver stiffness measurement; FIB‐4, fibrosis‐4 index; APRI, AST platelet ratio index.

Bold: p< 0.05.

**Figure 3:**
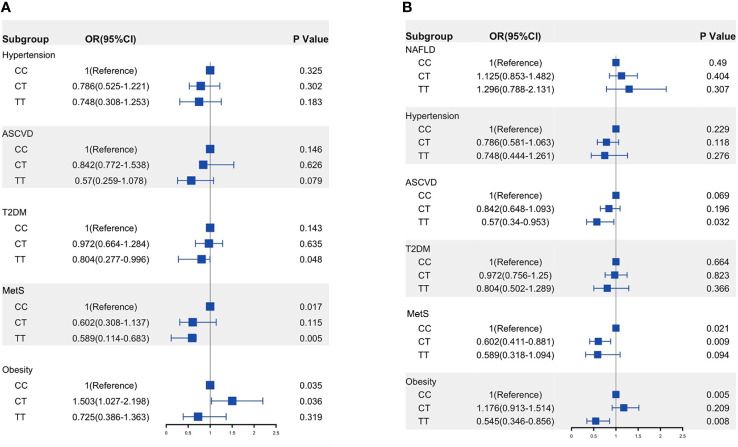
Risk of ASCVD and metabolic traits in the NAFLD patients **(A)** and whole population **(B)** according to MOBAT7 rs641738 C→T allele groups. OR was calculated using multivariable analysis and adjusted by age, sex, and BMI. p< 0.05 versus CC genotype. ASCVD, atherosclerotic cardiovascular disease; T2DM, type 2 diabetes; MetS, metabolic syndrome; NAFLD, non-alcoholic fatty liver disease.

The demographic and clinical characteristics of NAFLD patients according to C→T genotypes are listed in **Supplementary Table S1**. In multivariate logistic regression analysis, adjusting for demographics and anthropometrics (age, sex, and BMI), the TT genotype had a significantly lower risk of T2DM (OR:0.804, 95%CI: 0.277–0.996, p= 0.048) and MetS (OR: 0.590, 95%CI: 0.114–0.683, p= 0.005) when compared to the CC genotype (**Figure 3A**).

### Comparison of characteristics according to MBOAT7 rs641738 genotype and gender within NAFLD group

Gender is an important modified factor of NAFLD. Gender differences in clinical characteristics are listed in **Supplementary Table S2**. Male individuals were likely to have higher ALT and FBS levels and lower TC, HDL, and LDL levels; there were more patients with significant fibrosis and T2DM and fewer MetS patients.

To further investigate this observation, we compared the characters according to the genotypes in male and female patients separately. In male NAFLD patients, T allele carriage was associated with significantly higher levels of ALT, FINS, WHR, and significant fibrosis than non-T allele carriage (p=0.005, 0.041, 0.009, and 0.017, respectively, **Table 3**). However, in female NAFLD groups, the indicators were similar between the two genotypes. Although T allele carriage group demonstrated a higher level of TC (p=0.043, **Supplementary Table S3**) than non-T allele carriage, the difference disappeared after adjusting lipid-lowering agents usage.

**Table 3:** Comparison of clinical characteristics in different MBOAT7 rs641738 genotypes in male NAFLD patients.

Male NAFLD	CC (N=79)	CT+TT (N=71)	p-value
Age (year)	68.00 (66.00–70.00)	69.00 (67.00–74.00)	0.120
Weight (kg)	77.06 ± 9.74	77.45 ± 10.31	0.816
BMI (kg/m^2^)	27.25 ± 2.94	27.25 ± 2.88	0.990
Waist circumference (cm)	95.00 (89.00–100.00)	95.00 (89.00–100.00)	0.676
Hip circumference (cm)	103.00 (98.00–109.00)	101.00 (96.00–105.00)	0.212
Waist-to-hip ratio	0.92 (0.90–0.95)	0.94 (0.91–0.96)	**0.009**
ASCVD, n (%)	30 (37.70)	25 (34.80)	0.726
Hypertension, n (%)	71 (89.60)	58 (82.20)	0.149
MetS, n (%)	73 (92.40)	59 (83.10)	0.080
T2DM, n (%)	44 (55.70)	38 (53.50)	0.789
Obesity, n (%)	56 (70.90)	56 (78.60)	0.261
Lipid lowering agent, n (%)	39 (49.37)	15 (21.13)	**0.001**
ALT (U/L)	20.00 (15.00–26.00)	23.50 (18.00–29.00)	**0.005**
AST (U/L)	17.00 (14.00–21.00)	19.00 (15.75–23.00)	0.098
TG (mmol/L)	1.65 (1.06–2.02)	1.56 (1.17–2.07)	0.726
TC (mmol/L)	4.27 ± 0.85	4.44 ± 1.07	0.283
HDL (mmol/L)	1.00 ± 0.21	1.03 ± 0.19	0.202
LDL (mmol/L)	2.92 ± 0.77	3.06 ± 0.95	0.347
hs-CRP (mg/L)	1.41 (0.70–2.45)	1.80 (0.80–2.95)	0.433
FBS (mmol/L)	6.96 (6.04–8.80)	6.68 (5.92–8.17)	0.318
FINS (mIU/L)	10.57 (7.49–13.72)	12.86 (8.77–20.86)	0.041
HOMA-IR	3.36 (2.59–4.41)	3.70 (2.67–6.80)	0.091
HbA1c (%)	6.40 (5.90–7.90)	6.30 (5.95–7.30)	0.874
CAP (dB/m)	291.68 ± 47.72	295.08 ± 43.87	0.679
LSM (kPa)	4.90 (4.30–5.85)	5.10 (4.13–6.48)	0.658
≥8.2 Kpa, n (%)	2 (2.53)	11 (15.49)	**0.005**
FIB-4	1.32 (1.05–1.58)	1.41 (1.05–1.62)	0.484
APRI score	0.21 (0.15–0.27)	0.23 (0.16–0.30)	0.185
>0.5, n (%)	0 (0)	4 (1.90)	0.103

BMI, body mass index; WHR, Waist-to-hip ratio; ASCVD, atherosclerotic cardiovascular disease; MetS, metabolic syndrome; T2DM, type 2 diabetes; ALT, alanine aminotransferase; AST, aspartate aminotransferase; TG, total triglyceride; TC, total cholesterol; HDL, high‐density lipoprotein; LDL, low‐density lipoprotein; FBS, fast blood sugar; FINS, fast insulin; HOMA‐IR, homoeostatic model assessment of insulin resistance; CAP, controlled attenuated parameter controlled; LSM, liver stiffness measurement; FIB‐4, fibrosis‐4 index; APRI, AST platelet ratio index.

Bold: p< 0.05.

### Comparison of ASCVD prevalence and metabolic trait in MBOAT7 rs641738 C→T genotypes within the whole population

NAFLD always shares multiple manifestations of MetS, such as T2DM, hyperlipidemia, obesity, and hypertension (12, 25). Genetic epidemiology may be helpful to unveil the biological pathways that relate NAFLD to MetS and ASCVD. Thus, we compared ASCVD prevalence and metabolic traits among the three MBOAT7 genotypes.

In the whole population, TT carriage had fewer MetS, obesity, and ASCVD when compared to CC carriage (**Supplementary Table S4**). After adjusted by age, sex, and BMI, TT carriage showed a negative association with ASCVD (OR: 0.570, 95%CI: 0.340–0.953, p=0.032) and obesity (OR: 0.545, 95%CI: 0.346–0.856, p=0.008). Unfortunately, we did not find an association between MBOAT7rs641738 and NAFLD, and an increase in the minor T allele did not increase the risk of developing NAFLD (**Figure 3B**).

### Serum ANGPTL3 level in MBOAT7 C→T genotypes within the whole population

**Figure 4:**
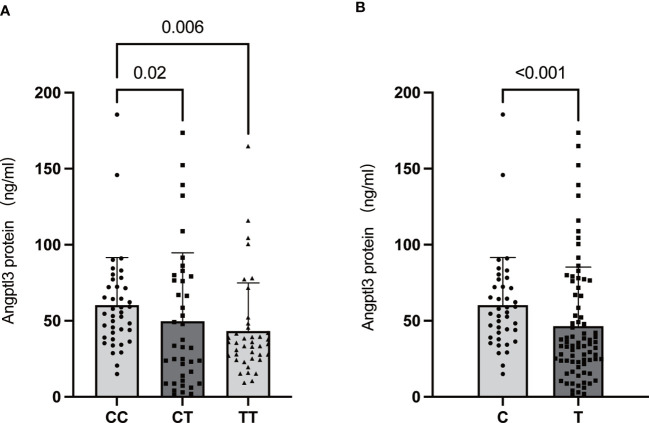
Association of the MBOAT7 rs641738 genotype (CC: homozygotes for the C allele; CT: heterozygotes; TT: homozygotes for the T allele) **(A)** and genotype carriage (C: homozygotes for the C allele; T: T allele carrier) **(B)** with serum ANGPTL3 levels in the total population. All data are shown as the mean ± SD. P<0.05.

ANGPTL3 has been identified as an important regulator of lipoprotein metabolism and is related to the risk of ASCVD (26). As shown above, the MBOAT7 rs641738 TT genotype was found to be associated with a reduced risk of ASCVD. Furthermore, the livers of MBOAT7^Δhep^ mice showed increased expression of ANGPTL3, a protein implicated in lipoprotein metabolism (18). Based on the above, we proposed that ANGPTL3 might be one of the molecular links between MBOAT7 rs641738 driven-NAFLD and ASCVD. As shown in **Figure 4**, serum ANGPTL3 level decreased paralleled from CC, CT, to TT genotype. The level in the TT genotype (43.27 ± 5.00 ng/ml) was significantly lower than that in the CC genotype (60.28 ± 4.95 ng/ml, p= 0.02).

## Discussion

This was the first study on the significance of the MBOAT7 rs641738 variant in an elderly population-based cohort. We found that the MBOAT7 rs641738 T allele was associated with higher ALT and significant fibrosis mainly in elderly male NAFLD patients. Furthermore, the TT genotype was associated with a decreased risk of T2DM and MetS in NAFLD patients and with a decreased risk of ASCVD and obesity in the total population. In addition, serum ANGPTL3 levels were significantly lower in TT allele carriers compared with CC genotype carriers, suggesting that ANGPTL3 may be the key molecular link between MBOAT7 variant-driven NAFLD and ASCVD.

Age is an important influencing factor for NAFLD. The prevalence of NAFLD peaks between 40 and 50 years old in men and 60 and 69 years old in women and then slightly decrease in people older than 70 years (27). The severity of NAFLD was also affected by age. A Hongkong study found that being older than 50 years old was one of the risk factors for NAFLD-related cirrhosis (28). Miyaaki et al. reported that being older than 60 years old was a risk factor for severe fibrosis (29). Although the subjects in our study were not a population with the highest prevalence rate of NAFLD, they might have additional advantages: (1) the severity of NAFLD seemed to peak in elders; (2) metabolic-associated diseases, such as T2DM and ASCVD, were highly prevalent in the population and facilitate the observation of their correlation with MBOAT7 rs641738 variants. Therefore, the elderly seemed to be better subjects to evaluate the association of gene variants with metabolic disease. As shown in **Table 1**, the prevalence rates of ASCVD and T2DM were as high as 35.28% and 41.58%.

NAFLD is a disease with high heterogeneity in which genetic predisposition plays an important role (30). Some studies have found several genes to be the major genetic determinants of NAFLD, including PNPLA3, TM6SF2, HSD17B13, and GCKR(31, 32). These gene variants combined with metabolic factors, such as obesity or diabetes, were proven to promote the progression of NAFLD by aggravating liver steatosis, inflammation, fibrosis, and hepatocarcinoma (30, 33). As mentioned above, MBOAT7 rs641738 C>T was an important inheritable factor in NAFLD incidence and progression (34–38). However, the relationships were mainly found in European descendants and not in Asians(Teo et al.; 17, 39–43). Only one study discovered that rs641738 C>T increased NAFLD occurrence in Chinese with an average age of 63 years old, and the frequency of the T allele was 43.42%. However, there was no relationship between the variant and fibrosis in the population (44). Different from the results, we found that the MBOAT7 rs641738 T allele promoted NAFLD inflammation and fibrosis but not NAFLD onset. As we all know, the effect of gene variants was not comparable in different ethnic groups due to the diversity of frequencies, penetrance, and relative risk. The frequency of the T allele in our cohort was 46.36%, which was higher than other Asian cohorts. In one Japanese cohort, two Korean cohorts, and one Taiwan children cohort, the T allele frequencies were 22.00% (39), 38.20% (43), 38.50% (40), and 19.60% (41), respectively. In European descents, the frequency was as high as 58.20% 16). Obviously, the higher prevalence of the variant facilitated the discovery of the effect of the variant.

The MBOAT7 gene encodes an acyltransferase that specifically esterifies arachidonic acid-CoA to lysophosphatidylinositol (LPI), thereby producing the main molecular species of phosphatidylinositol (PI) in the cell membrane (45). Thangapandi et al. (18) discovered that MBOAT7 deletion in hepatocytes can lead to the disruption of the intracellular PI side-chain remodeling pathway, which in turn promoted the pathological progression of liver fibrosis by upregulation of ECM genes in a high-fat, methionine-low, choline-deficient diet-fed mouse model of NAFLD. In addition, imbalance in LPI 18:0 levels have also been shown to be associated with the presence of fibrosis in human MBOAT7 rs641738 TT genotype carriers and in MBOAT7^Δhep^ mouse (18). The treatment of obese mice with exogenous LPI 18:1 promoted the expression of pro-fibrotic genes in the liver, and LPI lipids increased hepatic inflammation and fibrotic gene expression in an MBOAT7-dependent manner (46). Furthermore, circulating LPI levels have also been demonstrated to be significantly higher in patients with liver fibrosis compared to healthy individuals (46). The above-mentioned studies also demonstrated that MBOAT7-LPI-PI plays a key role in the development of fibrosis. NAFLD is an obvious sexual dimorphic disease (47, 48). The prevalence and severity of NAFLD are increasing in men more than in reproductive-age women. However, the incidence rate and severity of NAFLD were comparable or even worse than in men after menopause (49). However, NAFLD in female patients were all relatively mild compared to that in male patients in this study, characterized by lower ALT levels and less significant fibrosis. The effect of SNP is also different in male and female patients. A study showed that PNPLA3 rs738409 polymorphism was closely related to alcoholic liver disease in Chinese Han men (50). Gender differences in the MBOAT7 rs641738 variant have not been reported before. In our cohort, the associations of the T allele with ALT and fibrosis were only found in male NAFLD patients.

In our study, the association of the rs641738 variant with higher serum HDL was found but not TC and LDL-C. In a Spanish study, MBOAT7 variants were found to be associated with higher TC, LDL, and TG (51). Data from a genome-wide association study (852,409 participants) found that rs641738 variants were associated with higher TC only in Caucasian populations. A meta-analysis (17) showed that rs641738 variants were associated with lower TG and HDL only in Caucasian populations (17). We speculated that there were two reasons for the opposite results. First, the characteristics of the subjects were different in the studies. Second, there were more than 100 genes related to lipid metabolism. Thus, the relationship between a single gene and blood lipids was influenced by other factors, which were not taken into consideration collectively.

NAFLD was closely correlated with T2DM, and the relationship was recently proved to be influenced by SNPs. Moon et al. (52) discovered that a PNPLA3 polymorphism confers lower susceptibility to the incident of T2DM in NAFLD patients. In the NAFLD cohort of our study, the multivariant analysis showed that rs641738 T allele decreased the risk of T2DM. Such a correlation also resulted in the decrease in MetS in rs641738 T allele carriage. Previous research demonstrated that HDL reduce blood glucose levels through various mechanisms, such as promoting insulin secretion from pancreatic β (53) and enhancing glucose uptake into skeletal muscle by activating the AMPK signaling pathway (54). Clinical studies have also shown an association between lower levels of circulating HDL particles and T2DM, insulin resistance, and impaired glucose tolerance. (55). Femlak et al. found that low HDL-cholesterol (HDL-c) levels were an independent risk factor for type 2 diabetes and the development of microvascular complications of diabetes (56). In another study, the authors found a 50% decrease in both HDL_2_ cholesterol and protein levels in obese individuals by comparing HDL concentrations in 68 age-matched obese individuals with controls (57). The above study showed that HDL levels were significantly and negatively associated with the progression of metabolic diseases. In this study, we found that TT genotype carriers had a significantly lower risk of MetS in the NAFLD population after adjusting for confounding factors, which may be associated with a high HDL level being found in this population. However, as the statistical significance of the T allele and T2DM tended towards borderline values, additional mechanistic research and prospective longitudinal studies are necessary to confirm these findings.

As aforementioned, several gene loci have been found to promote NAFLD progression and prevent ASCVD, such as PNPLA3 rs738409 and TM6SF2 rs58542926. MBOAT7 variants seemed to exhibit similar effects according to our findings. A similar result was reported in a prospective follow-up study carried out in China Changfeng society. They found that the MBOAT7 T allele decreased the mortality rate of cardiovascular disease together with PNPLA3 and TM6SF2 gene variants (44). Two other findings might support such a correlation in our study. One finding was that TT variants were associated with less obesity in the whole population; the other was its association with fewer MetS and T2DM in NAFLD patients. No other clinical study or meta-analysis came to the same conclusion as us and the Changfeng study (12, 58). Whether such an association also had ethnic differences needs to be further confirmed in more studies.

A lower level of serum ANGPTL3 has been shown to decrease the risk of ASCVD by regulating serum TG. In an analysis of 13,102 patients with cardiovascular disease, carriers with loss-of-function mutations in ANGPTL3 had an approximately 40% lower risk of cardiovascular disease than non-carriers (59). In addition, Thangapandi et al. demonstrated that ANGPTL3 mRNA levels increased significantly in the liver of MBOAT7 knockout mice (18). Therefore, we supposed that ANGPTL3 may be a key regulatory molecule linked to MBOAT7 rs641738-related NAFLD and ASCVD. The hypothesis that serum levels of ANGPTL3 were significantly decreased in the TT group compared to the CC and CT groups was verified. ANGPTL3 is mainly synthesized by hepatocytes and then cleaved by PCSK9 to generate an N-terminal coiled-coil region and a C-terminal fibrinogen-like domain, which could be detected in the serum. MBOAT7 is a protein located on the hepatocyte membrane. MBOAT7 rs641738 variant decreased protein level and may interfere with syntheses or secretion of ANGPTL3 protein. The underlying mechanism needs to be further elucidated through additional *in vitro* and *in vivo* studies.

Several limitations should be noted in our study. First, the sample size was relatively small and with less opportunity to get more results. Second, the analysis was designed for the NAFLD study, so the relationship with ASCVD needs confirmation in specially designed research. Third, only one SNP was analyzed in the study; the confounders such as PNPLA3 SNP were not adjusted.

In conclusion, the rs641738 variant near MBOAT7 was found to promote inflammation and fibrosis, particularly in male patients, and decrease the risk of T2DM and metabolic traits in NAFLD patients. In addition, it was related to decreased ASCVD risk in the whole population. These results will be meaningful to better understand the pathogenesis of NAFLD and identify distinct phenotypes of the disease, which will benefit for individualized management.

## Data availability statement

The datasets presented in this study can be found in online repositories. The names of the repository/repositories and accession number(s) can be found below: https://figshare.com/, https://figshare.com/articles/dataset/MBOAT7_rs641738_C_T_is_associated_with_NAFLD_progression_and_decreased_ASCVD_risk_in_elder_Chinese_population/21506427.

## Ethics statement

The study protocol was approved by the individual Ethics Review Committee of Beijing Youan Hospital (IRB approval number [2020]-233). The patients/participants provided their written informed consent to participate in this study.

## Author contributions

XX, HX and XL participated in the acquisition, analysis, and interpretation of data; prepared the manuscript; and had equal contribution to the study. SZ, GW, and LZ reviewed the literature and built the databases. ZC, and LQ contributed to the analysis and interpretation of the data and provided important scientific input. XD and YL statistically analyzed the data and wrote the first draft of the manuscript. JZ and YZ supervised the whole study. All authors collaboratively discussed key decisions throughout the course of the review, provided critical feedback on preliminary manuscript and interpretation of results. All authors contributed to the article and approved the submitted version.
