# Supplementary material for: MBOAT7 rs641738 (C>T) is associated with NAFLD progression in men and decreased ASCVD risk in elder Chinese population
Source: Front Endocrinol (Lausanne). 2023 Jun 22;14:1199429. doi: 10.3389/fendo.2023.1199429 (PMC10324031; doi:10.3389/fendo.2023.1199429)
Supplement: Supplementary file 1 [file Table_1.docx]

Supplementary Material

**MBOAT7 rs641738 (C>T) is associated with NAFLD progression in male and decreased ASCVD risk in elder Chinese population**

**Authors**

Xiaoyi Xu^#1,3^, Hangfei Xu^#1,3^, Xiaohui Liu^#1^, Shuang Zhang^1,2^, Zhenhuan Cao^1^, Lixia Qiu^1^, Xiaofei Du^1^, Yali Liu^1^, Gang Wang^2^, Li Zhang^2^, Yang Zhang*^1,3^, Jing Zhang*^1^

^1^The Third Unit, The Department of Hepatology. Beijing Youan Hospital, Capital Medical University, Beijing, China

^2^Menkuang Hospital, Beijing Jingmei Group General Hospital

^3^Beijing Institute of Hepatology, Beijing, China

# Xiaoyi Xu, Hangfei Xu and Xiaohui Liu contributed equally to this work and shared first authorship

*** Correspondence author**

Jing Zhang, MD

The Third Unit, Department of Hepatology, Beijing Youan Hospital, Capital Medical University. No.8, Youwai Xiotutiao Street, Fengtai District, Beijing, China. 100069

Fax number :86-10-63056962

Mobile:13391859683

E-mail: zjyouan@ccmu.edu.cn

ORCiD number: 0000-0002-3082-8330

Yang Zhang, PHD

Beijing Institute of Hepatology

No.8, Youwai Xiotutiao Street, Fengtai District, Beijing, China. 100069

Fax number: 86-10-63057513

Mobile:13701385265

E-mail: yang518@mail.ccmu.edu.cn

ORCiD number: 0000-0001-7462-2935

| NAFLD | | C:C（N=333） | | C:T (N=256) | | T:T (N=49) | | p value | |
| --- | --- | --- | --- | --- | --- | --- | --- | --- | --- |
| Age（year） | | 69.00(66.00~72.00) | | 69.00(67.00~73.00) | | 69.00(67.00~73.00) | | 0.542 | |
| Male, n (%) | | 79(23.72) | | 60(23.44) | | 11(22.45) | | 0.980 | |
| Weight（Kg） | | 67.30(60.80~74.50) | | 67.10(61.60~75.10) | | 66.60(58.45~73.00) | | 0.655 | |
| BMI (Kg/m2) | | 26.50(24.50~28.80) | | 27.10(25.20~28.90) | | 26.30(24.40~28.35) | | 0.100 | |
| Waist circumference (cm) | | 90.00(85.00~97.00) | | 90.00(86.00~95.00) | | 90.00(84.00~95.00) | | 0.387 | |
| Hip circumference (cm) | | 100.00(95.00~105.50) | | 100.00(95.00~104.75) | | 98.00(93.00~105.00) | | 0.194 | |
| WHR | | 0.90(0.88~0.93) | | 0.91(0.89~0.94) | | 0.90(0.88~0.94) | | 0.149 | |
| ASCVD, n (%) | | 116(34.90) | | 94(36.90) | | 11(22.40) | | 0.156 | |
| Hypertension, n (%) | | 273(82.00) | | 203(79.40) | | 39(80.30) | | 0.700 | |
| MetS, n (%) | | 313(94.00) | | 234(91.40) | | 40(81.60)^b^ | | 0.024 | |
| T2DM, n (%) | | 159(47.70) | | 118(46.10) | | 16(32.70)^b^ | | 0.141 | |
| Obesity, n (%) | | 233(70.0) | | 200(78.00)^a^ | | 31(63.30) | | **0.027** | |
| Lipid lowering agent, n (%) | 124(37.24) | | 81(31.64) | | 11(22.45) | | 0.078 | |  |
| ALT (U/L) | | 19.00(15.00~25.00) | | 20.00(16.00~27.00) | | 18.00(14.00~25.50) | | 0.101 | |
| AST (U/L) | | 18.00(14.00~22.00) | | 18.00(15.00~22.50) | | 17.00(14.00~21.50) | | 0.828 | |
| TG (mmol/L) | | 1.56(1.12~2.05) | | 1.52(1.10~2.02) | | 1.74(1.25~2.26) | | 0.278 | |
| TC (mmol/L) | | 4.59(3.87~5.38) | | 4.79(4.09~5.61) | | 5.11(4.25~5.95)^b^ | | **0.012** | |
| HDL (mmol/L) | | 1.09(0.95~1.23) | | 1.11(0.98~1.26) | | 1.12(0.99~1.33) | | 0.072 | |
| LDL (mmol/L) | | 3.15(2.50~3.90) | | 3.28(2.65~4.08) | | 3.56(2.88~4.30)^b^ | | **0.018** | |
| hs-CRP (mg/L) | | 1.50(0.80~2.60) | | 1.60(0.93~2.90) | | 1.80(0.78~3.81) | | 0.427 | |
| FBS (mmol/L) | | 6.50(5.90~7.85) | | 6.55(5.89~7.92) | | 6.43(5.71~6.99) | | 0.259 | |
| FINS (mIU/L) | | 10.67(7.51~14.04) | | 11.67(8.55~18.07)^a^ | | 11.17(7.40~18.23) | | **0.049** | |
| HOMA-IR | | 3.25(2.27~4.51) | | 3.61(2.56~6.05) | | 3.25(2.04~5.30) | | 0.114 | |
| HbA1c (%) | | 6.30(5.90~7.30) | | 6.20(5.90~7.10) | | 6.20(5.88~6.50) | | 0.206 | |
| CAP (dB/m) | | 295.43$\pm40.97$ | | 299.44$\pm$40.96 | | 288.47$\pm$39.50 | | 0.205 | |
| LSM (kPa) | | 5.00(4.10~6.10) | | 4.90(4.20~6.35) | | 5.30(4.10~6.50) | | 0.958 | |
| ≥8.2 Kpa, n (%) | | 30(9.00) | | 25(9.80) | | 6(12.80) | | 0.775 | |
| FIB-4 | | 1.29(1.01~1.67) | | 1.24(0.92~1.60) | | 1.30(1.05~1.61) | | 0.622 | |
| APRI score | | 0.20(0.15~0.27) | | 0.19(0.15~0.27) | | 0.20(0.14~0.27) | | 0.976 | |
| >0.5, n (%) | | 6(1.80) | | 13(5.08) | | 3(6.12) | | 0.052 | |

**Supplementary table 1.** **Comparison of clinical characters among different MBOAT7rs641738 genotypes in the NAFLD group**

BMI, body mass index; WHR, Waist-to-hip ratio; ASCVD, atherosclerotic cardiovascular disease; MetS, metabolic syndrome; T2DM, type 2 diabetes; ALT, alanine aminotransferase; AST, aspartate aminotransferase; TG, total triglyceride; TC, total cholesterol; HDL, high‐density lipoprotein; LDL, low‐density lipoprotein; FBS, fast blood sugar; FINS, fast insulin; HOMA‐IR, homoeostatic model assessment of insulin resistance; CAP, controlled attenuated parameter controlled; LSM, liver stiffness measurement; FIB‐4, fibrosis‐4 index; APRI, AST platelet ratio index

a: CC versus CT genotype p<0.05

b: CC versus TT genotype p<0.05

**Supplementary Table 2.** **Comparison of clinical characteristics between male and female NAFLD patients**

| NAFLD | Male（N=150） | Female（N=488） | p value |
| --- | --- | --- | --- |
| Age（year） | 68.00（66.00~72.00） | 69.00（67.00~73.00） | 0.333 |
| Weight（Kg） | 76.60（69.75~84.00） | 64.60（59.73~71.05） | **<0.001** |
| BMI (Kg/m^2^) | 27.00（24.90~29.35） | 26.70（24.70~28.68） | 0.117 |
| Waist circumference (cm) | 95.00(89.00~100.00) | 90.00(84.00~94.00) | **<0.001** |
| Hip circumference (cm) | 102.00(97.75~107.00) | 99.00(95.00~103.75) | **<0.001** |
| Waist-to-hip ratio | 0.93(0.91~0.95) | 0.90(0.88~0.93) | **<0.001** |
| ASCVD, n (%) | 54(36.30) | 167(34.20) | 0.689 |
| Hypertension, n (%) | 123(82.20) | 389(79.70) | 0.538 |
| MetS, n (%) | 132(88.00) | 455(93.20) | **0.039** |
| T2DM, n (%) | 82(54.70) | 211(43.20) | **0.014** |
| Obesity, n (%) | 112(74.50) | 352(72.10) | 0.571 |
| Lipid lowering agent, n (%) | 54(36.30) | 162(33.20) | 0.526 |
| ALT (U/L) | 21.00(17.00~27.50) | 19.00(15.00~25.00) | **0.001** |
| AST (U/L) | 18.00(15.00~22.00) | 18.00(15.00~22.25) | 0.947 |
| TG (mmol/L) | 1.61（1.13~2.03） | 1.54（1.12~2.06） | 0.875 |
| TC (mmol/L) | 4.29（3.62~5.07） | 4.89（4.14~5.66） | **<0.001** |
| HDL (mmol/L) | 1.00（0.87~1.12） | 1.13(1.00~1.27) | **<0.001** |
| LDL (mmol/L) | 2.90(2.30~3.69) | 3.39(2.68~4.09) | **<0.001** |
| hs-CRP (mg/L) | 1.50(0.71~2.60) | 1.60(0.83~2.70) | 0.505 |
| FBS (mmol/L) | 6.82(5.97~8.44) | 6.45(5.80~7.63) | **0.006** |
| FINS (mIU/L) | 11.21(8.42~16.24) | 11.18(7.88~15.71) | 0.721 |
| HOMA-IR | 3.54(2.64~5.18) | 3.30(2.25~5.00) | 0.238 |
| HbA1c (%) | 6.35(5.90~7.48) | 6.20(5.90~7.00) | 0.150 |
| CAP (dB/m) | 295.00(271.50~324.50) | 299.00(270.00~324.75) | 0.600 |
| LSM (kPa) | 4.90（4.20~6.30） | 5.00（4.10~6.30） | 0.990 |
| ≥8.2 Kpa, n (%) | 13(8.70) | 48(9.90) | 0.670 |
| FIB-4 | 1.36(1.05~1.61) | 1.23(0.95~1.63) | 0.181 |
| APRI score | 0.22(0.16~0.28) | 0.19(0.14~0.26) | **0.009** |
| >0.5, n (%) | 4(2.70) | 18(3.70) | 0.549 |
| MBOAT7 rs641738 |  |  |  |
| CC | 79(52.70) | 254(52.00) | 0.980 |
| CT | 60(40.00) | 196(40.20) |  |
| TT | 11(7.30) | 38(7.80) |  |

BMI, body mass index; WHR, Waist-to-hip ratio; ASCVD, atherosclerotic cardiovascular disease; MetS, metabolic syndrome; T2DM, type 2 diabetes; ALT, alanine aminotransferase; AST, aspartate aminotransferase; TG, total triglyceride; TC, total cholesterol; HDL, high‐density lipoprotein; LDL, low‐density lipoprotein; FBS, fast blood sugar; FINS, fast insulin; HOMA‐IR, homoeostatic model assessment of insulin resistance; CAP, controlled attenuated parameter controlled; LSM, liver stiffness measurement; FIB‐4, fibrosis‐4 index; APRI, AST platelet ratio index

**Supplementary Table 3. Comparison of clinical characteristics different MBOAT7 rs641738 genotypes in female NAFLD patients**

| Female NAFLD | CC（N=254） | CT＋TT（N=234） | p value |
| --- | --- | --- | --- |
| Age（year） | 69.00(66.00~73.00) | 67.00(69.00~73.00) | 0.707 |
| Weight（Kg） | 64.60(59.28~70.53) | 64.70(60.18~71.60) | 0.748 |
| BMI (Kg/m^2^) | 26.30(24.50~28.60) | 27.10(24.90~28.73) | 0.162 |
| Waist circumference (cm) | 90.00(84.00~95.00) | 90.00(84.00~93.00) | 0.539 |
| Hip circumference (cm) | 100.00(95.00~105.00) | 98.00(95.00~103.00) | 0.239 |
| Waist-to-hip ratio | 0.90(0.87~0.93) | 0.90(0.88~0.92) | 0.465 |
| ASCVD, n (%) | 86(34.00) | 81(34.50) | 0.860 |
| Hypertension, n (%) | 202(79.60) | 186(79.70) | 0.991 |
| MetS, n (%) | 240(94.50) | 215(91.90) | 0.252 |
| T2DM, n (%) | 115(45.30) | 96(41.00) | 0.344 |
| Obesity, n (%) | 177(69.70) | 175(74.80) | 0.209 |
| Lipid lowering agent, n (%) | 85(33.46) | 77(32.91) | 0.896 |
| ALT (U/L) | 19.00(15.00~24.25) | 19.00(15.00~25.00) | 0.754 |
| AST (U/L) | 18.00(14.00~23.00) | 17.00(15.00~22.00) | 0.488 |
| TG (mmol/L) | 1.55(1.12~2.07) | 1.54(1.13~2.06) | 0.948 |
| TC (mmol/L) | 4.77(4.00~5.56) | 5.00(4.27~5.81) | **0.043** |
| HDL (mmol/L) | 1.12(0.99~1.24) | 1.16(1.01~1.31) | 0.056 |
| LDL (mmol/L) | 3.29(2.56~4.00) | 3.50(2.77~4.20) | 0.055 |
| hs-CRP (mg/L) | 1.52(0.80~2.68) | 1.60(1.00~2.95) | 0.299 |
| FBS (mmol/L) | 6.46(5.79~7.64) | 6.44(5.80~7.61) | 0.784 |
| FINS (mIU/L) | 10.73(7.50~14.35) | 11.47(8.26~17.50) | 0.096 |
| HOMA-IR | 3.23(2.19~4.66) | 3.49(2.33~5.44) | 0.207 |
| HbA1c (%) | 6.30(5.90~7.18) | 6.20(5.85~6.90) | 0.135 |
| CAP (dB/m) | 296.00(267.50~324.75) | 302.00(272.00~324.75) | 0.559 |
| LSM (kPa) | 5.00(4.10~6.25) | 5.00(4.10~6.30) | 0.962 |
| ≥8.2 Kpa, n (%) | 27(10.6) | 21(9) | 0.540 |
| FIB-4 | 1.28(1.00~1.67) | 1.21(0.91~1.60) | 0.233 |
| APRI score | 0.20(0.15~0.26) | 0.19(0.14~0.25) | 0.337 |
| >0.5, n (%) | 6(2.4) | 12(5.1) | 0.105 |

BMI, body mass index; WHR, Waist-to-hip ratio; ASCVD, atherosclerotic cardiovascular disease; MetS, metabolic syndrome; T2DM, type 2 diabetes; ALT, alanine aminotransferase; AST, aspartate aminotransferase; TG, total triglyceride; TC, total cholesterol; HDL, high‐density lipoprotein; LDL, low‐density lipoprotein; FBS, fast blood sugar; FINS, fast insulin; HOMA‐IR, homoeostatic model assessment of insulin resistance; CAP, controlled attenuated parameter controlled; LSM, liver stiffness measurement; FIB‐4, fibrosis‐4 index; APRI, AST platelet ratio index

**Supplementary Table 4. Comparison of ASCVD prevalence rate and metabolic trait among *MBOAT7* rs641738 genotypes in whole population**

| Total | C:C（N=605） | C:T(N=435) | T:T (N=88) | p value |
| --- | --- | --- | --- | --- |
| Age（year） | 69.00(67.00~74.00) | 69.00(67.00~74.00) | 69.00(67.00~73.00) | 0.920 |
| Male, n (%) | 168(27.80) | 125(28.70) | 23(26.10) | 0.868 |
| Weight（Kg） | 64.60(58.50~71.90) | 65.45(59.28~72.53) | 63.25(55.78~69.38) | 0.060 |
| BMI (Kg/m2) | 25.50(23.73~27.80) | 25.90(23.78~28.10) | 24.60(22.90~27.10)b | 0.018 |
| Waist circumference (cm) | 88.00(83.00~95.00) | 88.00(84.00~93.00) | 86.00(81.00~90.00)b | 0.046 |
| Hip circumference (cm) | 98.00(94.00~103.00) | 98.00(94.00~102.00) | 96.00(92.00~100.00)b | 0.019 |
| Waist-to-hip ratio | 0.90(0.87~0.93) | 0.90(0.88~0.93) | 0.90(0.88~0.93) | 0.126 |
| NAFLD,n (%) | 333(55.00) | 256(58.90) | 49(55.70) | 0.467 |
| ASCVD, n (%) | 228(37.70) | 148(34.00) | 23(25.60) | 0.080 |
| Hypertension, n (%) | 477(78.80) | 329(75.60) | 63(72.10) | 0.215 |
| MetS, n (%) | 528(87.30) | 361(83.00) | 69(78.40)b | 0.033 |
| T2DM, n(%) | 255(42.10) | 183(42.10) | 32(36.40) | 0.576 |
| Obesity, n (%) | 352(58.20) | 270(62.10) | 38(43.20)b | 0.004 |
| Lipid lowering agent, n (%) | 226(37.36) | 137(31.49) | 25(28.41) | 0.068 |
| ALT (U/L) | 17.50(13.00~23.00) | 19.00(15.00~24.00)a | 18.00(14.00~23.00) | 0.014 |
| AST (U/L) | 17.00(14.00~21.00) | 18.00(15.00~22.00) | 17.00(14.00~21.75) | 0.399 |
| TG (mmol/L) | 1.38(0.96~1.89) | 1.35(0.97~1.89) | 1.46(1.07~2.05) | 0.428 |
| TC (mmol/L) | 4.59(3.89~5.40) | 4.84(4.09~5.64)a | 4.92(4.10~5.56)b | 0.008 |
| HDL(mmol/L) | 1.12(0.97~1.28) | 1.15(0.99~1.32)a | 1.14(1.02~1.32) | 0.036 |
| LDL (mmol/L) | 3.15(2.51~3.91) | 3.33(2.70~4.09)a | 3.42(2.75~3.96) | 0.023 |
| hs-CRP (mg/L) | 1.20(0.60~2.40) | 1.30(0.72~2.42) | 1.38(0.68~2.58) | 0.442 |
| FBS (mmol/L) | 6.31(5.69~7.53) | 6.23(5.68~7.34) | 6.21(5.71~7.05) | 0.742 |
| FINS(mIU/L) | 9.25(6.13~12.80) | 9.82(6.48~14.62) | 8.67(6.70~14.47) | 0.104 |
| HOMA-IR | 2.73(1.76~3.93) | 2.86(1.76~4.63) | 2.62(1.76~4.59) | 0.248 |
| HbA1c(%) | 6.10(5.80~6.90) | 6.10(5.80~6.90) | 6.20(5.73~6.70) | 0.940 |
| CAP(dB/m) | 275.00(238.00~310.00) | 277.00(238.75~313.00) | 270.00(244.00~307.00) | 0.769 |
| LSM(kPa) | 4.60(3.80~5.80) | 4.60(3.80~5.90) | 4.80(4.10~6.10) | 0.576 |
| ≥8.0 Kpa, n (%) | 39（6.45） | 32(7.36) | 8(9.09) | 0.616 |
| FIB-4 | 1.34(1.06~1.70) | 1.31(0.97~1.66) | 1.36(1.03~1.66) | 0.382 |
| APRI score | 0.20(0.15~0.26) | 0.20(0.15~0.27) | 0.20(0.15~0.27) | 0.978 |
| >0.5, n (%) | 11(1.80) | 17(4.00) | 3(3.40) | 0.117 |

BMI, body mass index; WHR, Waist-to-hip ratio; ASCVD, atherosclerotic cardiovascular disease; MetS, metabolic syndrome; T2DM, type 2 diabetes; ALT, alanine aminotransferase; AST, aspartate aminotransferase; TG, total triglyceride; TC, total cholesterol; HDL, high‐density lipoprotein; LDL, low‐density lipoprotein; FBS, fast blood sugar; FINS, fast insulin; HOMA‐IR, homoeostatic model assessment of insulin resistance; CAP, controlled attenuated parameter controlled; LSM, liver stiffness measurement; FIB‐4, fibrosis‐4 index; APRI, AST platelet ratio index

a: CC versus CT genotype p<0.05

b: CC versus TT genotype p<0.05

**
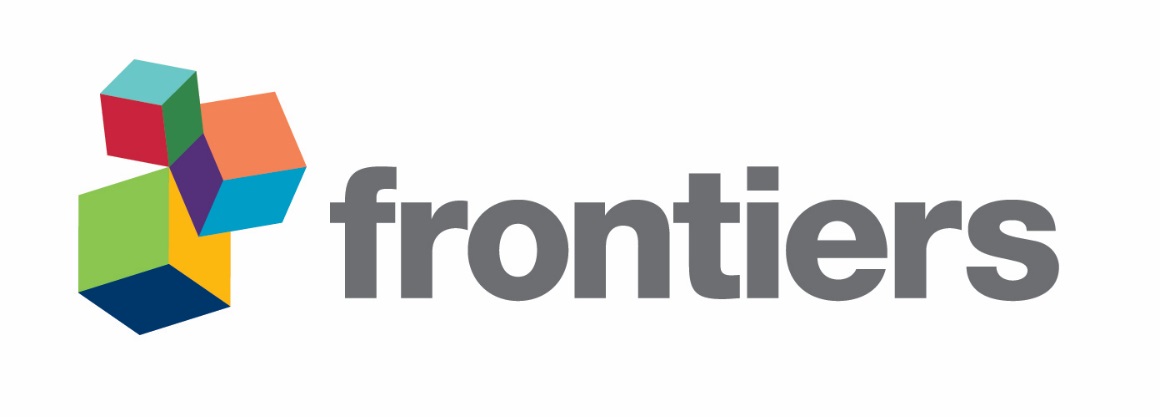
**
